# Sexual dimorphism of the mandibular conformational changes in aging human adults: A multislice computed tomographic study by geometric morphometrics

**DOI:** 10.1371/journal.pone.0253564

**Published:** 2021-06-22

**Authors:** Leonor Costa Mendes, Julien Delrieu, Claudia Gillet, Norbert Telmon, Delphine Maret, Frédéric Savall

**Affiliations:** 1 Laboratoire d’Anthropobiologie AMIS, UMR 5288 CNRS, Université Paul Sabatier, Toulouse, France; 2 UFR d’Odontologie de Toulouse, Toulouse, France; 3 Service de Médecine Légale, CHU Toulouse Rangueil, Toulouse, France; Università di Roma, ITALY

## Abstract

The aging process has an impact on mandibular bone morphology and can therefore affect shape sexual dimorphism. Understanding the effect of senescence on mandibular shape changes is particularly important to correctly estimate the sex of an individual and predict age-related conformational modifications. The purpose of this study was to assess age-related changes in mandibular shape and sexual dimorphism. The study sample comprised 160 Multi Slice Computed Tomography examinations of individuals aged 40 to 79 years. Geometric morphometric analysis of fourteen osteometric landmarks was used to examine sexual dimorphism and patterns of mandibular shape variation with age. Results showed that mandibular sexual dimorphism of shape remained significant with aging. Conformational changes occurred between 50 and 70 years and were different for male and female individuals. Females presented earlier and more marked age-related shape changes than males. These observations suggest that mandibular senescence is a sexually dimorphic process since its onset, rate, and the areas subjected to conformational changes differ from male to female individuals. Senescence-related changes present substantial variability, and further investigation is required to determine precisely the age that marks their onset.

## Introduction

The cephalic extremity is a valuable element for adult sexual estimation. Both the facial skeleton and the mandible are highly dimorphic structures and are used in anthropological, archaeological and forensic studies with good classification accuracy rates [1, 2].

It is presently known that certain areas of the cephalic extremity undergo resorption with aging, and that among these areas, some present a stronger predisposition to conformational changes [3, 4]. The morphology of the cranium and upper facial skeleton is affected by the aging process [5], but these changes are particularly visible in the mid and lower face, and have different effects in male and female individuals. The maxilla, and particularly the pyriform region, are subjected to bone remodeling and resorption, as are the superomedial and inferolateral aspects of the orbital rim, leading to a retrusion of the lower midfacial skeleton [6–10]. On the mandible, morphological changes are also visible with advancing age [11, 12]. However, there appears to be a lack of consensus regarding the trend of mandibular shape variation with aging [13–15].

Following a study by Enlow et al., the assumption that the mandible expands continuously with aging was widely accepted [16]. This was further supported by subsequent investigations, who related that midfacial and mandibular growth continues, although at different rates, through late adulthood [7, 11]. Several authors have indeed reported that mandibular height, width and length had a tendency to increase in older individuals [11, 12, 15, 17]. However, these studies were either composed of small samples [11, 16, 17] or their younger group comprised individuals who had not attained full skeletal maturity [12, 15, 17], inadvertently giving a result of mandibular growth between young and older age groups. Shaw et al., using metric measurements in mature Caucasian individuals, found a decrease in mandibular body height and length, as well as ramus height, with advancing age [18]. The tendency for a senescence-related mandibular atrophy was confirmed by Toledo Avelar et al. [14]. Nonetheless, it appears that certain areas are more subjected to age-related remodelling, such as the symphysis region, the alveolar ridge, the ramus, including the condyle and coronoid process, and the gonial area [3, 12, 14, 16, 18, 19].

The impact of aging in sex estimation accuracy of the facial skeleton has been observed by a few authors with qualitative [20] and quantitative (measurement) [21] methods. There appears to be a general increase in sexual classification accuracy of the cranium and mandible with advancing age, and these changes begin to emerge between 40 and 50 years of age [5, 20–22]. Although aging of specific features of the skeleton and soft tissues of the face has been studied, in particular with regard to facial plastic and reconstructive surgery [23, 24], there is a paucity of information regarding the evolution of sexual dimorphism of the mandible with advancing age, and the effect of age-related changes in mandibular conformational dimorphism remains unclear. Several authors have reported that the most dimorphic mandibular traits are the gonial area, followed by the condylar position, the coronoid process shape and the inclination of the alveolar process in the symphysis area [25–27]. Since senescence has an impact on these areas, we can assume that it could have an effect on mandibular sexual dimorphism.

Knowledge of senescence-related conformational changes with regard to sexual dimorphism is particularly important to correctly estimate the sex of an individual, and can be especially valuable to improve facial age progression techniques for the identification of long-missing persons and facial reconstructions [13].

Two-dimensional measurements, as used in traditional quantitative methods, cannot be used to quantify complex three-dimensional changes of the aging bone [28, 29]. Three-dimensional (3D) geometric morphometrics (GMM) is an accurate and reliable method for sex estimation allowing the study of morphological differences between individuals, particularly nonmetrical features not easily described by linear measurements [27, 30]. The main advantages of this method are that it enables differentiation of variability due to both size and shape, as well as the appreciation of shape conformational changes without subjective bias [1].

The primary aim of our study was to determine whether there exist age-related changes in mandibular shape. Our secondary aim was to assess if mandibular sexual dimorphism of conformation remains significant throughout middle and old age, following the analysis of these shape changes. We addressed the hypothesis that males and females present different mandibular conformational changes with aging. For this purpose, we used geometric morphometric analysis to study the mandibular shape variation of Multi Slice Computed Tomography (MSCT) examinations.

## Materials and methods

### Reference sample

Craniofacial multi-slice computed tomography (MSCT) scans of adult patients, performed between august 2014 and may 2019, were collected from the Toulouse University Hospital radiology archives (CHU Rangueil, Toulouse). The MSCT examinations were requested in a clinical context of vascular disease. Patients with a known history of facial pathology or trauma were excluded. A total of 160 MSCT scans were included, corresponding to 80 male and 80 female individuals, aged 40 to 79 years. Each age group presented an homogeneous and sequential distribution, and the mean age of the male and female individuals was 59,5 years and standard deviation (SD) 11,6 years (Table 1).

**Table 1:** Sample size and age group composition.

Age group (years)	Sample size	
	Male	Female
**40–44**	10	10
**45–49**	10	10
**50–54**	10	10
**55–59**	10	10
**60–64**	10	10
**65–69**	10	10
**70–74**	10	10
**75–79**	10	10

### Data collection and post-processing

MSCT images were obtained using a picture archiving and communication system (PACS, McKesson Medical Imaging Group, Richmond, BC, Canada) used by our institution. Examinations were performed on a Sensation 16 Scanner (General electric medial system, Erlangen, Germany) with 16 × 1.5 mm collimation. The image matrix was 512 × 512 pixels, axial slices’ thickness was 0.6 mm and voxel size was 15 μm. The CT scans were saved as Digital Imaging and COmmunications in Medicine (DICOM) files. Post-processing was performed using Horos™ Medical Imaging software v.3.3 64-bit and consisted in the application of a bone filter to improve spatial resolution.

### Landmark selection and positioning

**Fig 1:**
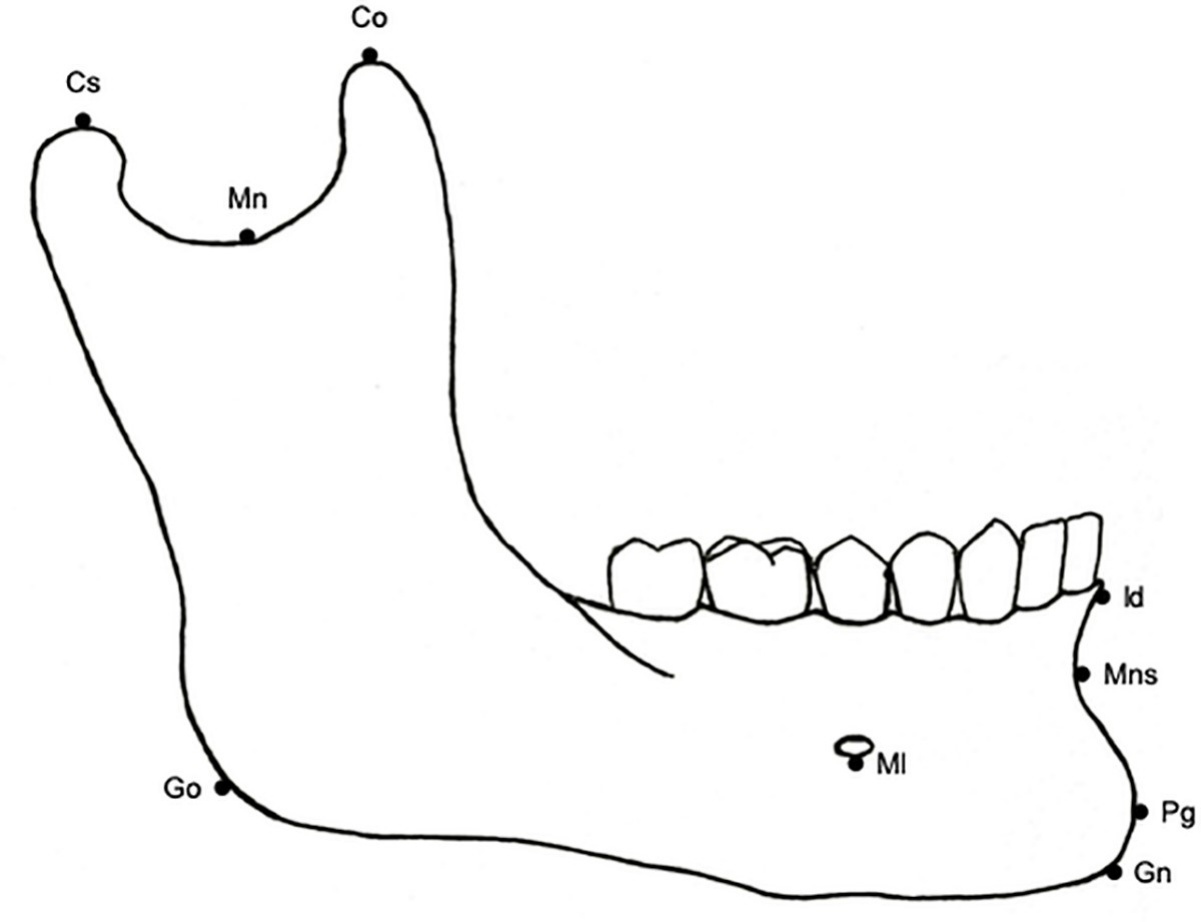
Mandibular landmarks used in the present study. Landmark’s definitions are listed in Table 2.

Based on standard anthropometric techniques and on the literature, 14 osteometric landmarks were selected on the mandible [25, 31, 32] (Fig 1). The chosen landmarks were type I landmarks (points whose structural location presents a strong homology and that are therefore easy to identify repetitively, *e*.*g*. juxtaposition of tissues, intersection of three sutures, foramina), or type II landmarks (points defined by geometric criteria, *e*.*g*. the tip of a structure, the point of maximum curvature along a structure) [30]. Type I and type II landmarks are used in classic osteometric methods for bone sexing thus allowing comparisons with previously published results. The landmarks were selected to represent the overall shape of the symphyseal region, the ramus and the inferior border of the mandible.

**Table 2:** Mandible landmark definitions.

Landmark	Name	Definition
**Cs**	Condylion superior	The most superior point on the mandibular condyle
**Mn**	Mandibular notch	The most inferior point on the mandibular notch
**Co**	Coronion	The most superior point on the coronoid process
**Go**	Gonion	The most lateral external point of the junction of the horizontal and ascending rami of the lower jaw
**Ml**	Mentale	The most inferior point at the margin of the mandibular mental foramen
**Id**	Infradentale	The midline point of the superior tip of the septum between the mandibular central incisors
**Mns**	Mandibular symphysis	The deepest point of the mandibular symphysis curvature (between the infradentale and pogonion landmarks)
**Pg**	Pogonion	The most anterior midline point on the chin of the mandible
**Gn**	Gnathion	The most inferior midline point on the mandible

**Fig 2:**
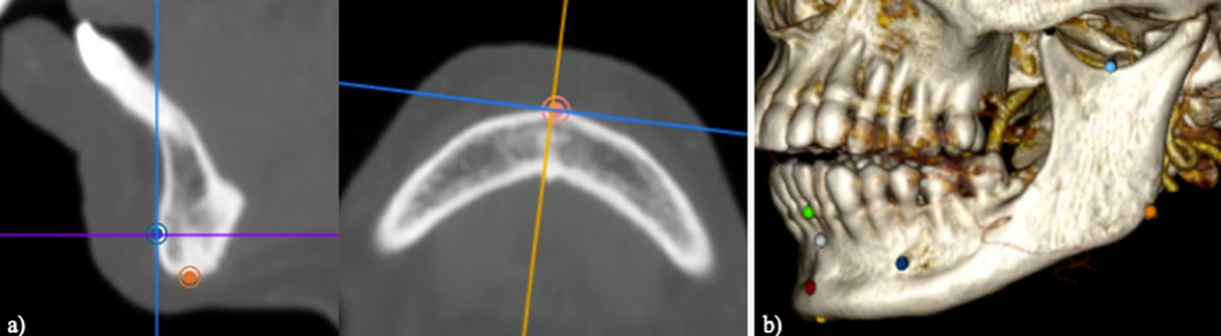
Landmark placement in Horos DICOM Viewer. (a) Multiplanar reconstruction on Horos showing the placement of the *Pogonion (Pg)* landmark. (b) 3D volume rendering displaying the mandibular landmarks as coloured dots.

The landmarks were positioned using Horos™ DICOM Viewer (v.3.3 64-bit). The point tool was used to place the landmarks on the mandibular MSCT reconstructions using the multi-planar reconstruction (MPR) mode (Fig 2A). Their correct location was then validated on the three-dimensional volume rendering mode (Fig 2B). The corresponding 3D coordinates (*x*, *y*, and *z*) for each landmark were subsequently recorded. All the landmarks used are listed in Table 2.

### Morphological 3D analysis

Calculations and statistical analyses were carried out using R 3.0.2 software version 3.6.1^©^ [33], the *shapes* (v1.2.5; Dryden, 2019) [34], *geomorph* (v3.2.1; Adams, Collyer & Kaliontzopoulou, 2020) [35] packages, as well as the *mshape*, *procGPA*, *procD*.*lm*, *procdist* and *CVA* functions. The data and R code that support the findings of this study are openly available in Open Science Framework at http://doi.org/10.17605/OSF.IO/KB3HG [36].

#### Repeatability

Geometric morphometric methods rely on the accurate identification and placement of landmarks on biological specimens. A landmark presenting a strong homology is a landmark that can repeatedly be identified with precision and that can provide information about shape changes along any of the three axes. In order to validate the accuracy and reproducibility of the landmark placement method used in this study, the assessment of inter and intra-observer agreement is desirable [37], so that any researcher can repeat the presented protocol. Intra-rater and inter-rater reliability of landmark selection was tested using the Generalizability Theory (GT) as described by Ercan et al. [38]. In GT, the reliability for relative (norm-referenced) interpretations is referred to as the generalizability (G) coefficient (landmark pairs-by-rater-by-subject, l x r x s). Two observers performed the landmark selection twice: the first observer (LCM) carried out a second analysis of 40 randomized specimens from the sample (20 males and 20 females), 1 month after the first analysis, and a second observer (JD) completed an analysis of the same 40 specimens. G-coefficients between the two landmark sets from the same rater (intra-observer) and from the two different raters (inter-observer) were calculated. A G-coefficient close to 1 indicates high intra/inter-rater reliability [38–40].

#### Geometric morphometrics

Landmark-based geometric morphometric analyses were used to address the following two questions: the study of age-related variations of mandibular conformation and the estimation of mandibular sexual dimorphism.

The first step consisted in removing shape-unrelated variations in the sample (such as differences in scale, position and orientation) using generalized Procrustes analysis (GPA) [41] (*shapes* and *geomorph* packages, and *procGPA* function). GPA superimposes specimen landmark coordinates by translating them to a common origin, rotating them according to a least squares criterion aimed at minimizing Procrustes distances, and scaling them to unit centroid size until the coordinates of corresponding points align as closely as possible [30]. Centroid size is obtained by calculating the square root of the sum of the squared Euclidean distances of all landmarks to the centroid of the configuration [30]. This procedure allows the study of mandibular conformational differences independently of size.

To assess age-related conformation variations of the mandible and estimate the period in which mandibular senescence-related shape changes start to become visible, we tested age as a separate variable in different age groups. As our sample was composed of individuals presenting a balanced distribution between 40 and 79 years, we arbitrarily established three cut-offs at 55, 60 and 65 years, and performed a Procrustes ANOVA with permutation procedures to test shape differences between the younger and older age cohorts for both males and females. The statistical significance of differences was tested with Goodall’s *F*-test, using Procrustes residuals (*procD*.*lm* function). A Principal Component Analysis (PCA) of the landmark coordinates was subsequently performed for the whole sample, to summarize the main trends in shape variation between sexes as well as the younger and older age cohorts. In addition, the mandibular aging rate within the sampled age period was also estimated separately for males and females [42]. As our sample is relatively small, we performed a multivariate regression of mandibular shape on age in a moving window of 20 individuals and estimated the average amount of mandibular shape change in units of Procrustes distance per year.

Finally, to compare the differences in mandibular shape between age cohorts, a consensus configuration, or mean shape configuration, was produced and represented graphically as wireframes, that is as lines between landmarks (*mshape* function).

To examine mandibular shape sexual dimorphism and determine whether shape differed between sexes and age groups, sex, age and size were studied as separate variables. Following a common GPA on all data, a regression between shape and sex with age as covariate was then performed, as well as a regression between shape and size with sex as covariate (*procD*.*lm* function).

A GPA followed by a Procrustes ANOVA with permutation tests was used to evaluate shape differences between the sexes in two age groups spanning a twenty-year range (40–59 years and 60–79 years). The magnitude of sexual dimorphism was assessed by calculating Procrustes distances and Mahalanobis distances between the sexes (*mshape* and *procdist* functions). Procrustes distance provides a measure of the differences in the positions of the landmarks in two shapes [30, 43], whereas Mahalanobis distance measures the distances between group centroids based on a scale adjusted to the within-group variance [44]. Goodall’s *F*-test, performed on Procrustes residuals, tested the statistical significance of differences between sexes for the younger and older age groups. To compare the differences between male and female configurations, a consensus configuration was produced and represented graphically as wireframes.

### Ethical considerations

All data and images used in this study were recorded anonymously, only sex and age were recorded at the time of the computed tomography (CT) acquisition. According to French law, the results of medical imaging examinations may be used retrospectively without the patient’s consent when these examinations have been carried out for clinical purposes and when they have been recorded anonymously (article 40–1, law 94–548 of July 1, 1994).

## Results

### Repeatability

All 14 mandibular landmarks presented a strong inter and intra-observer agreement. The G-coefficient for both intra and inter-observer reliability was G = 0.94, indicating high reliability of the landmark selection.

### Geometric morphometrics

#### Age-related changes in mandibular shape

To evaluate age-related conformation variations of the mandible, male and female individuals were studied separately. Permutation tests between age cohorts revealed statistical shape changes for males and females under and over 65 years. In the female sample, results were also statistically significant when the cut-off was placed at 55 and 60 years (Table 3).

**Table 3:** Male and female age-related mandibular conformational changes.

Differences between age cohorts Cut-off	Goodall’s F	*p*-value
**Males**		
**55 years** (40–54 vs 55–79 years)	1.0951	0.344
**60 years** (40–59 vs 60–79 years)	1.6681	0.084
**65 years** (40–64 vs 65–79 years)	2.2903	0.016
**Females**		
**55 years** (40–54 vs 55–79 years)	3.261	0.004
**60 years** (40–59 vs 60–79 years)	3.8611	0.001
**65 years** (40–64 vs 65–79 years)	3.2856	0.002

The male and female samples are divided into two age cohorts, with a cut-off at 55, 60 and 65 years. Shape comparisons are performed between the younger and older age cohorts for males and females separately.

**Fig 3:**
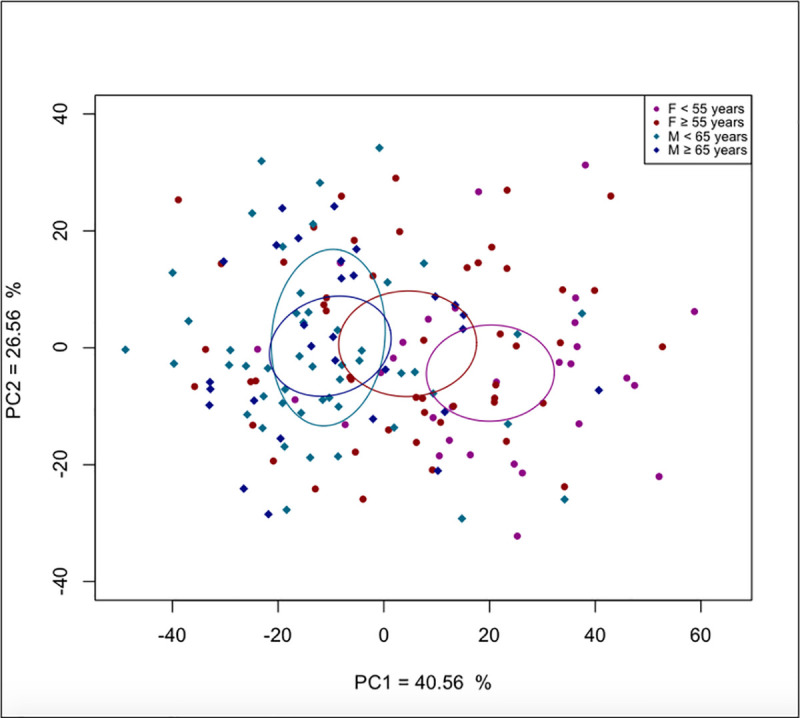
Principal Component Analysis of male and female mandibular shape. Each sex is divided into two age cohorts: 40–64 years vs. 65–79 years for males, and 40–54 years vs. 55–79 years for females. The effect of age on mandibular conformation is more marked for females.

For the Principal Components Analysis (PCA), male and female samples were divided into two age cohorts: individuals aged 40 to 54 versus 55 to 79 years for females, and individuals aged 40 to 64 versus 65 to 79 years for males. The first Principal Component axis (PC1) accounted for 40.56% of age-related shape variations. Although considerable variation in individual mandibular shape could be observed, female shape differences between the younger and older individuals were better discriminated than male’s along PC1 (Fig 3).

The results of the PCA seemed to indicate that female mandibular shape draws closer to male mandibular shape with aging. In order to support this hypothesis, Procrustes distances were computed between the four age cohorts (younger and older males and females). The results showed that the distance between old females and old males (0.0232; *p* = 0.012) is smaller than the distance between young females and young males (0.0296; *p* = 0.001). Furthermore, the distance between young females and old males (0.0371; *p* = 0.001) is greater than the distance between old females and young males (0.0269; *p* = 0.001).

**Fig 4:**
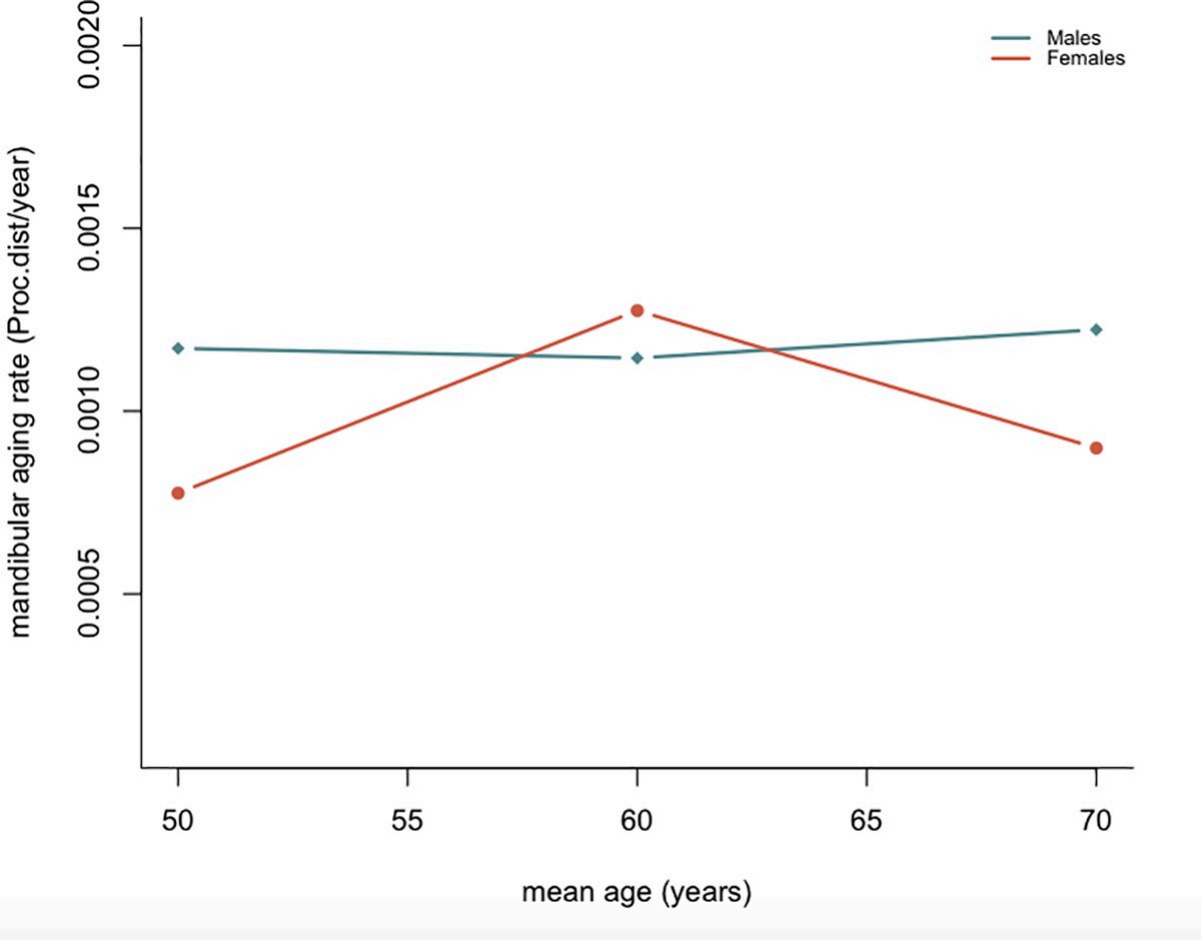
Male and female mandibular aging rates. An acceleration of female mandibular shape changes can be observed between 50–60 years. The rate of conformational changes is estimated in Procrustes distance / year.

Males displayed a relatively constant aging rate (the average amount of mandibular shape change per year). For females, however, an acceleration of the aging pattern could be observed from 50 years onwards, with a peak around 60 years of age (Fig 4).

Consensus configuration shapes were created for males and females. Based on previous results (Table 3), mean shape configurations of female individuals were compared before and after 55 years of age. For males, the cut-off was placed at 65 years.

**Fig 5:**
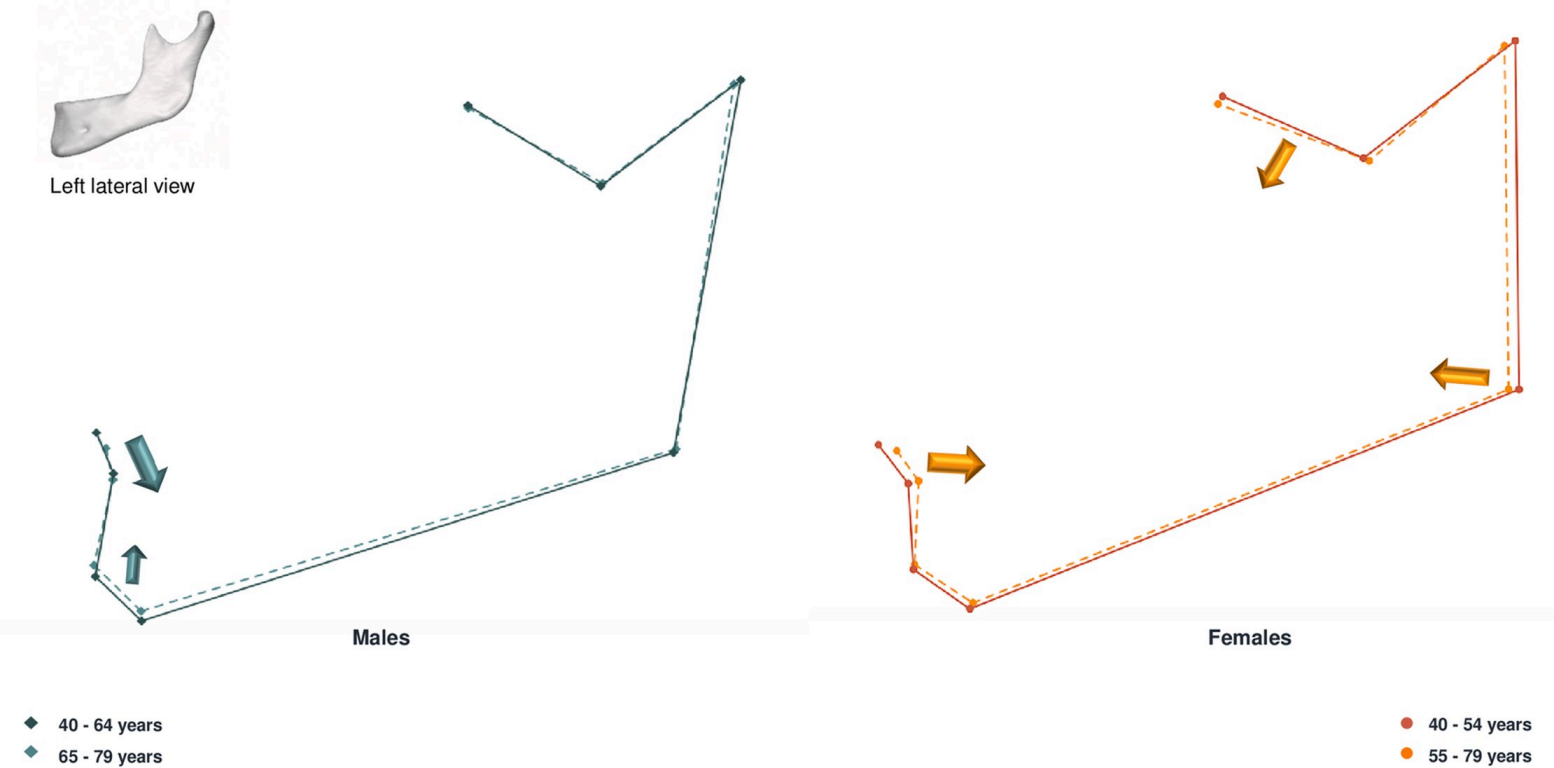
Lateral view of male and female mandibular shape variations with age. On the left: male mean shapes: 40–64 years (solid lines) vs 65–79 years (dashed lines). On the right: female mean shapes 40–54 years (solid lines) vs 55–79 years (dashed lines). The arrows represent the major directions and magnitude of changes in conformation with age.

**Fig 6:**
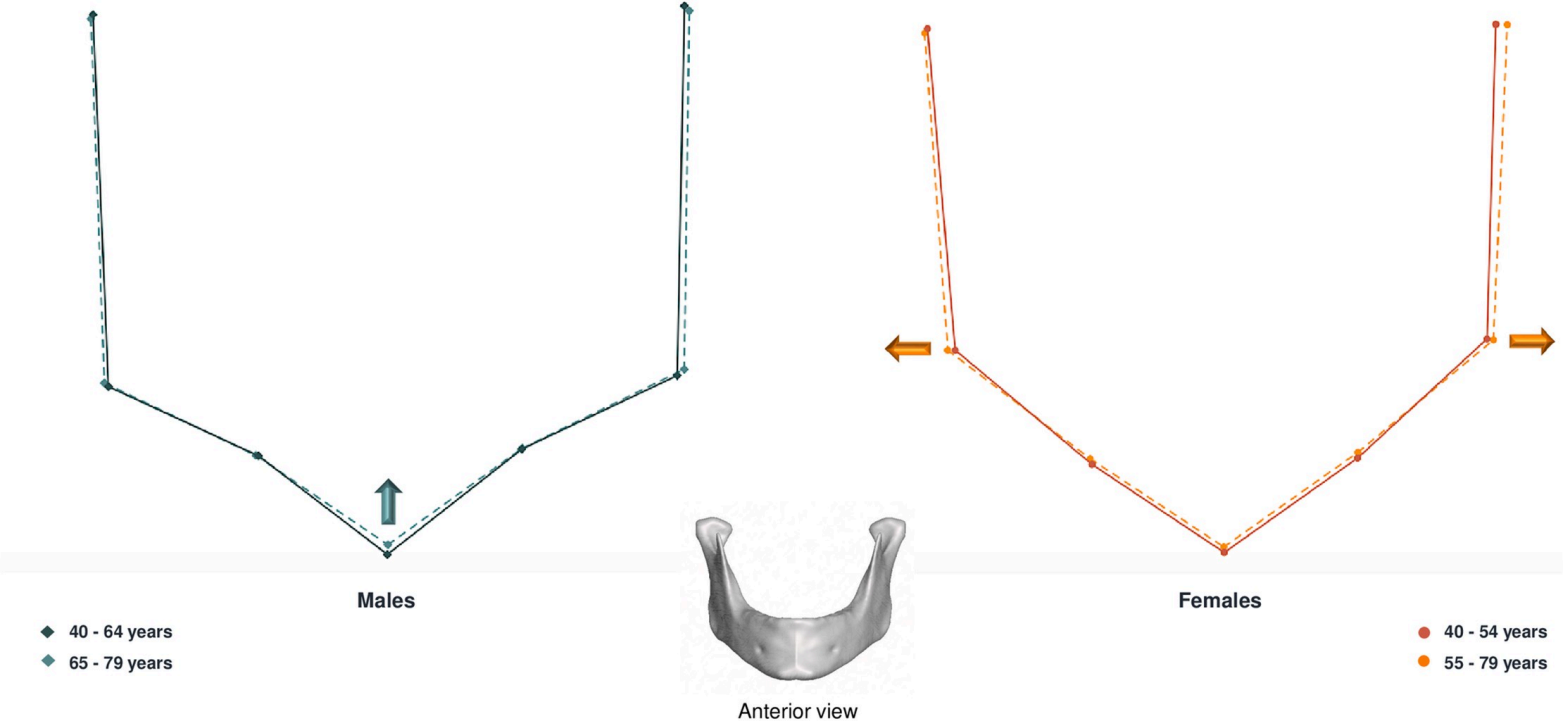
Anterior view of male and female mandibular shape variations with age. On the left: male mean shapes: 40–64 years (solid lines) vs 65–79 years (dashed lines). On the right: female mean shapes 40–54 years (solid lines) vs 55–79 years (dashed lines). The arrows represent the major directions and magnitude of changes in conformation with age.

Regarding sex-specific shape variations, both males and females exhibited marked conformational changes in the mandibular symphysis region (Figs 5 and 6). Men presented mostly vertical changes, the upper symphysis following a downward and posterior movement, while the gnathion moved upwards. Women presented vertical, lateral and sagittal changes with aging. The gonion moved laterally, upwards and forwards. There was a retrusion of the symphysis whereas the posterior border of the ramus moved in an anterior direction.

#### Mandibular shape sexual dimorphism

Both sex, age and size, as separate variables, had a statistically significant effect on mandibular shape (*p* < 0.001). However, the interactions of age and sex and size and sex were not statistically significant *(p* = 0.197) (Table 4).

**Table 4:** Effect of age, sex and interaction of sex, age and size on mandibular shape for the whole sample (n = 160).

Variables	Goodall’s F	*p*-value
Sex	5.1496	0.001
Age	4.4970	0.001
Size	7.4342	0.001
Sex: Age	1.3074	0.197
Size: Sex	0.7926	0.685

Procrustes distances, Mahalanobis distances and Goodall’s *F*-test revealed that sexual dimorphism of shape was statistically significant for the whole sample (*p* < 0.001), as well as the two age groups tested (Table 5).

**Table 5:** Analysis of shape sexual dimorphism for the whole sample (n = 160), and permutation tests between sexes by age groups.

	Mahalanobis distance	Procrustes distance	Goodall’s F	*p*-value
**F40-59 / M40-59**	3.2974	0.0282	3.9291	0.001
**F60-79 / M60-79**	2.4278	0.0246	2.9421	0.002
**Whole sample (F/M)**	2.0781	0.0159	5.0285	0.001

The sample is divided in two age groups (n = 40) both for males and females.

Shape differences were visualized by means of consensus configuration shapes, produced for males and females. The sample was divided in two age groups: 40 to 59 years and 60 to 79 years. The mean female and male mandibular shapes were then superimposed and sexual dimorphism was visualized as wireframe displacements between the younger and older age groups.

**Fig 7:**
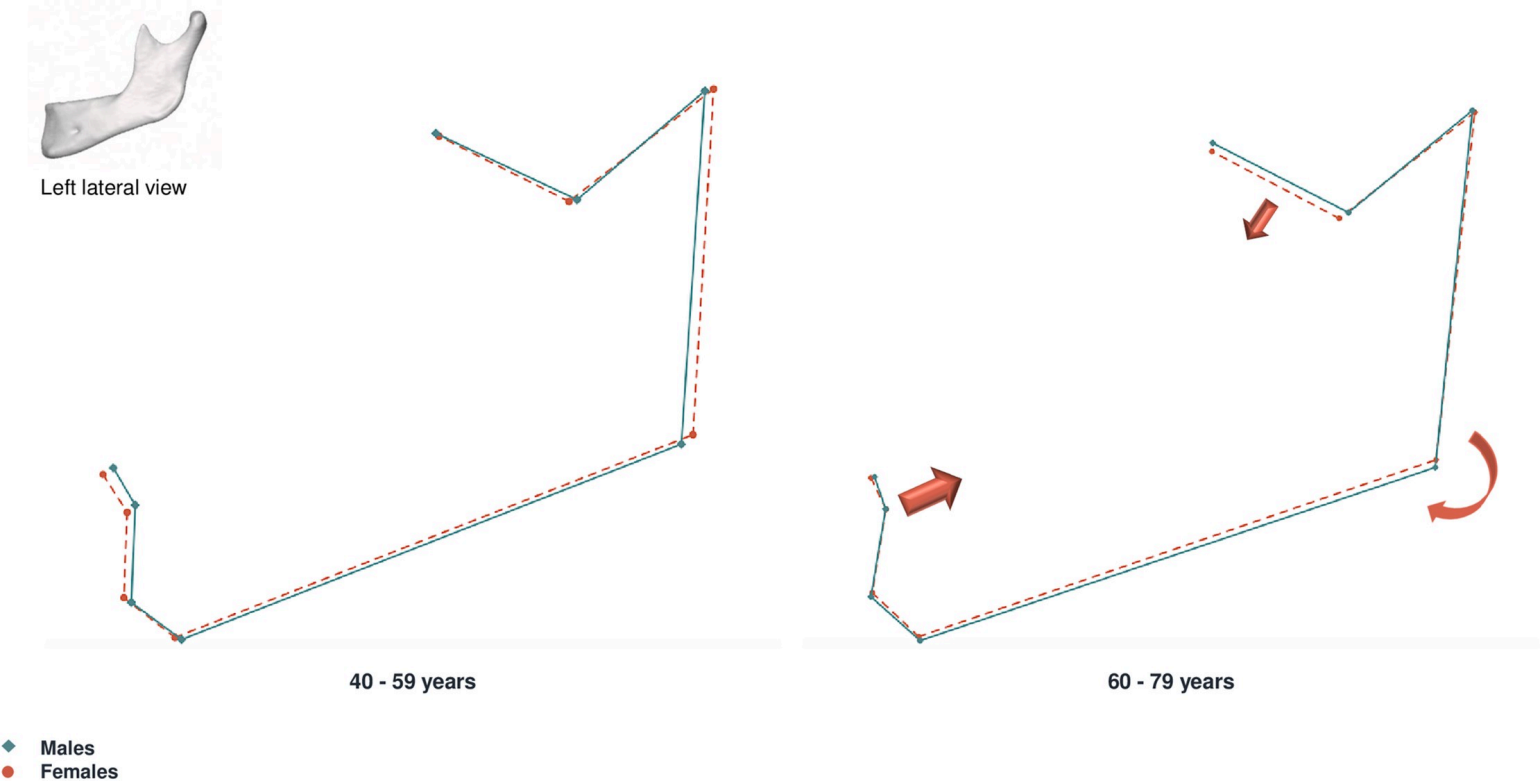
**Left lateral view of male (blue) and female (red) mean shape superimposition.** On the left: 40–59 year group. On the right: 60–79 year group. Males are represented as solid lines, females are represented as dashed lines. Arrows represent the main trend of variation for female individuals between age groups.

**Fig 8:**
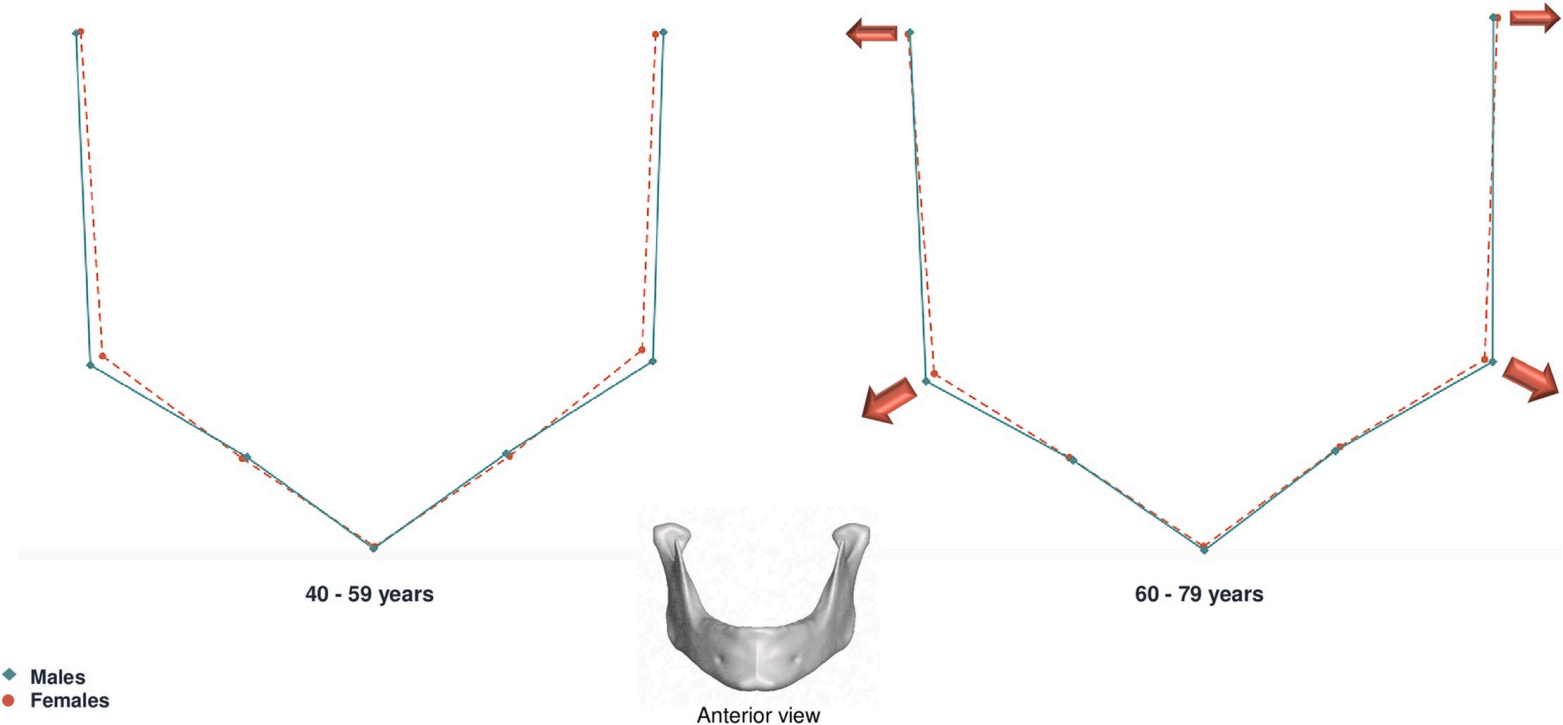
**Anterior view of male (blue) and female (red) mean shape superimposition.** On the left: 40–59 year group. On the right: 60–79 year group. Males are represented as solid lines, females are represented as dashed lines. Arrows represent the main trend of variation for female individuals between age groups.

Male and female mean shape superimpositions showed differences in the gonial region, the upper and posterior ramus region (coronoid, condyle and mandibular notch), and the mandibular symphysis (Figs 7 and 8). In the younger age group (40–59 years), shape differences between males and females were particularly marked in the symphysis area. Females presented more prominent symphysis in the younger age group, but the consensus configurations converged after 60 years. The gonion and condyle were more lateral in males than in females between 40 and 59 years but with advancing age this difference was less pronounced. Overall, the sexual differences observed in the younger group had a tendency to decrease after 60 years of age.

## Discussion

Earlier studies have established that the senescence process has an impact on mandibular bone morphology, and that certain areas are more subjected to age-related remodelling, such as the symphysis region, the alveolar ridge, the ramus and the gonial area [3, 12, 14, 16, 18, 19]. As these areas are the most dimorphic within the mandibular bone, there is probably an evolution of mandibular sexual dimorphism with aging.

The purpose of our work was therefore to analyze the nature of senescence-related shape changes both within and between sexes, and to assess if sexual dimorphism remained significant in the mandible despite these conformational changes, using geometric morphometrics.

Our results showed, by means of consensus configuration superimpositions, that conformational differences occur with age for both males and females (Figs 5 and 6). Male and female individuals presented marked conformational changes in the symphysis, gonial, coronoid and condylar areas. The global trend of variation followed an eccentric direction in the gonial area and a concentric direction in the symphysis, ramus and condylar regions. Both sexes presented an anterior and inferior resorption of the mandibular body, as found in previous studies [14]. The symphysis was the area where age-related shape changes were most visible. Several authors have found substantial conformational changes of the symphysis with aging. Shaw et al. described a decrease in mandible length leading to similar chin projection for men and women during the middle age years [18]. Toledo Avelar et al. also noted that the chin becomes more prominent, oblique and its height decreases in older individuals [14].

The analysis of shape variations with age for each sex revealed an earlier onset of changes for females, starting at 55 years, whereas for males significant conformational differences seemed to appear from 65 years onwards (Table 3). An earlier set up of the senescence process in women, as well as a faster mandibular aging rate, could account for the greater conformational changes observed in this sample (Fig 4). As the pace of age-related conformation changes appears to be slower for male individuals and they display a later onset of the senescence process, it is possible that marked configurational changes cannot be discerned in our sample, which is limited to individuals aged up to 79 years. Similar aging trajectories have been reported by Windhager et al., who observed a divergence in the facial aging pattern in women and men after menopause (around 50 years), as well as an earlier onset and higher pace of facial aging in females [42].

We know that other facial structures are subjected to earlier changes in women, particularly the midfacial skeleton [6, 24, 45]. Kahn & Shaw observed earlier and more substantial changes in females’ orbital aperture, glabellar and pyriform angles [6]. As in our study, women presented in general more extensive bone loss, with resorption starting between the young and middle age groups for females, and between the middle and old age groups for males. These observations indicate that the senescence process differs between male and female individuals, and increases inter-individual shape variability. However, it would also appear that after the onset of the aging process, intra-individual variability can be observed, since all facial structures are not affected simultaneously. Cotofana et al. found that the pace of age-related changes is not homogeneously distributed across the midfacial region: some bony structures present earlier changes than others [23]. In the mandible, it has been described that conformational changes in length start before changes in height [18].

Although senescence had an effect in all individuals, the nature of the shape changes was different in males and females. While the main conformational changes take place in the symphysis area for men, and are particularly marked the alveolar process, the whole mandible is subjected to shape alterations in women. As in men, the most affected area is the mandibular symphysis, in which a marked retrusion is observed after 55 years. Females also display a lateralization of the mandibular ramus, with the gonion and condyle moving outwardly, and an anterior displacement of the gonial and coronoid regions.

Previous studies have similarly found that age-related changes in the mandible differ from male to female individuals, and that the latter present more marked conformational changes [3, 4]. Pecora et al., in a longitudinal cephalometric study, noted that for men changes took mostly place on the anterior portion of the mandible, leading to increased chin prominence with aging. Women, on the other hand, presented more changes in the vertical dimension, with a posterior rotation of the mandible [12]. These observations indicate that the nature of age-related conformational changes is subjected to dimorphism, which corroborates our results.

When analysing mandibular sexual dimorphism in different age groups using geometric morphometrics we observed that sexual dimorphism of shape remained statistically significant (p<0.005) for all age groups (Table 5).

Former studies, using qualitative and quantitative methods, have reached similar conclusions [20, 28, 46]. It has been reported that aging can even have a positive impact on sex diagnosis of the cephalic extremity, with better prediction accuracies after the setting of age-related changes. Suazo and Matamala found an increase in the diagnostic performance of the morphological indicators used for sexual dimorphism of the skull and mandibles of older subjects [20]. Other studies observed an increase in sexual classification accuracy of the mandible [21] and cranium [5] for individuals aged over 40 years. For Gapert et al., accuracy rates in sex estimation of the foramen magnum region rose after 50 years of age, particularly in women [22]. However, most of these studies were based on metric measurements, and therefore considered mainly size rather than shape sexual dimorphism.

In our sample, although sex, age and size undoubtedly had an effect on mandibular shape, the interaction of sex and age was not statistically significant, implying that age did not enhance sexual dimorphism of shape (Table 4). The hypothesis that can be formulated from this observation is that male and female mandibular shapes move in the same direction with aging. This could mean that both sexes have a tendency towards masculinization or feminization. In light of the PCA results and Procrustes distances between age cohorts, it would appear that females move towards males with aging.

In addition, as males tend to present larger features than females, the interaction between size and sex was not statistically significant.

In order to investigate the evolution of shape differences between sexes we divided our sample in two age groups (40–59 years and 60–79 years) (Figs 7 and 8). Mean shape superimpositions between sexes showed that differences occurred with age, and were mainly located in the symphysis, gonial areas, as well as the upper ramus region (coronoid, condyle and mandibular notch). Differences in conformation are particularly marked before 60 years, with females presenting a more projected symphysis. Our results are in agreement with former studies, in which this region appears to be one of the most dimorphic in the mandible, with males presenting a higher, wider symphysis and less projected mental eminence than women [28, 47]. As we saw previously that women present greater shape changes, our results seem to indicate that the female configuration shape draws nearer to the male configuration shape with aging, and that sexual dimorphism of conformation declines with age (Figs 7 and 8). This is further supported by the fact that we found no significant correlation when studying the interaction of sex and age with shape (Table 4). Pessa et al., in a longitudinal study, also observed that shape differences between males and females tended to fade with advancing age [17].

In line with our first aim, our findings reveal that the senescence process leads to mandibular conformational changes with aging. We also confirm that sex-related differences in mandibular shape can be observed during the senescence process, allowing us to accept the hypothesis that males and females present different mandibular conformational changes with aging.

Furthermore, this study shows that the senescence process is not only subjected to variability between genders but also between individuals of the same sex. Aging is a process influenced by multiple external and internal factors, and presents therefore higher heterogeneity than growth and maturation. Mandibular morphology is greatly influenced by external factors such as hormones and muscular shape. The earlier start of conformational changes in women could be explained by the hormonal effect of menopause, since a decrease in hormone levels, particularly oestrogen, causes a stronger resorption of the facial bones, particularly the mandible [42, 48]. Although both sexes are affected by hormonal variations with advancing age, bone turnover increases rapidly in menopausal women, while in men this process is more gradual [48].

Among the factors that could account for interindividual differences during senescence are muscle function and edentulousness. On average, males produce greater muscle forces during mastication, which results in larger mandibles than females, and rougher surfaces of muscle attachments, especially at the coronoid process and gonion [25, 49, 50]. Bakke et al. have reported that bite force decreases significantly with age, and that this process starts earlier and is more pronounced in female individuals [49]. Chrcanovic et al. suggested that maintenance of greater masticatory muscle strength and larger muscles in males with age could explain the differences in mandibular conformation between sexes [51]. On the other hand, we know that tooth loss increases with advancing age [52], and this can also affect masticatory muscle function. Partially or totally edentulous individuals produce lower bite forces than fully dentate individuals due to loss of occlusal contacts [53]. This reduction in muscular activity can lead to mandibular conformational changes, particularly in the areas of masticatory muscle attachment such as the gonial and coronoid regions. In addition, tooth loss leads to resorption of the alveolar process, since its main function is to provide structural support for the dentition [16, 19]. In the anterior mandible, bone loss is vertical and horizontal, moving in a concentric direction, whereas the posterior mandible is mostly subjected to vertical resorption, and horizontal resorption occurs eccentrically [54]. Several authors have stated that dentition is the main determinant of mandibular morphology, since tooth loss affects particularly mandibular body height, length and gonial angle [15, 29, 51, 52, 54]. In our sample, most of the changes in males took place in the mandibular symphysis, more specifically in the alveolar process, while female individuals presented more widespread conformation variations. As information regarding tooth loss is missing in this sample, we can hypothesize that age-related changes in males could be predominantly derived from tooth loss while, in females, the effect of edentulousness could be less striking due to the influence of other factors such as a decrease in oestrogen levels.

Insight into the facial senescence process is paramount in order to accurately estimate an individual’s sex and predict age-related conformational changes. This can be particularly useful in the fields of facial reconstruction and recognition, as well as the identification of long-missing persons by improving facial age progression techniques [13].

The use of geometric morphometrics and MSCT examinations present several advantages in the study of facial skeletal aging and sexual dimorphism. GMM allows the study of shape-specific variations, encompassing complex three-dimensional changes of the aging bone [55]. The same landmarks as those employed in classic archaeologic and anthropometric methods can be used, thus allowing comparisons with previously published results and existing standards, and their collection is facilitated and more precise than with dry bones. Furthermore, MSCT and 3D landmark data enable the creation of facial virtual standards for both the facial skeleton and overlying soft tissues. Several studies have observed that there is a close link between facial soft tissue shape and underlying bone, and that skeletal aging has a direct impact on the cutaneous envelope [56–58]. Thus, accurate prediction of bone conformational changes with aging could allow the estimation of facial soft tissue shape.

In our sample, although mandibular shape changes were clearly visible with aging, we could not precisely determine the age that marked the start of the senescence process. This could be due to the fact that the number of studied subjects is not high enough. Considering that our sample size did not allow the comparison of shape differences at successive age bins as this would result in small samples and therefore loss of statistical power, we established three cut offs at 55, 60 and 65 years in order to study larger groups and target the time span in which age-related shape variations emerge. As our results showed that conformation variations appear to be significant between 50 and 70 years, a larger sample of individuals aged 50 to 70 years and divided into 5 or 10-year age bins would be necessary to refine these results. Another limitation of this study is the fact that the sample is limited to individuals aged up to 79 years. In the event that males present a later onset of the senescence process, the entirety of shape changes cannot be encompassed. Finally, other variables such as dental status should be studied in order to acquire a deeper understanding of mandibular age-related conformational changes.

## Conclusion

Our findings revealed that mandibular sexual dimorphism remains present throughout middle and old age. Mandibular senescence appears to be a sexually dimorphic process since its onset, pace, and the areas subjected to variation differ from male to female individuals. Female individuals present faster, more marked and extensive shape changes than males. The mandibular areas mostly affected by age-related variation are the symphysis, gonion and upper ramus. Senescence appears as a highly heterogeneous process, it is therefore difficult to determine with precision the age that marks its onset. In this study, age-related changes seem to occur between 50 and 70 years for males and females, and can be detected earlier in female individuals.
